# 

**Published:** 1887-02

**Authors:** 


### CONTENTS.

### COMMUNICATIONS.

Syphilis—Its Dangers and Results........................................ 57
Radical Cure of Alveolar Abscess by Injection of Gutta Percha Solution........................................ 59
Anæsthesia........................................ 61
Alveolar Abscess........................................ 73
Elementary Life........................................ 80
The Field of Operative Dentistry........................................ 85

### SELECTIONS.

Surgical Aphorisms........................................ 94
The Wonderful Drug Clerk........................................ 97
SALMAGUNDI........................................ 101

### EDITORIAL.

An Interesting Meeting........................................ 105
A Change of Position........................................ 107
Proceedings Cleveland Dental Society........................................ 108
A Special Request........................................ 108

